# Supplementary material for: LineageVAE: reconstructing historical cell states and transcriptomes toward unobserved progenitors
Source: Bioinformatics. 2024 Aug 22;40(10):btae520. doi: 10.1093/bioinformatics/btae520 (PMC11494380; doi:10.1093/bioinformatics/btae520)
Supplement: btae520_Supplementary_Data [file btae520_supplementary_data.zip › LineageVAE__OUP_General_Template (supplementary).pdf]

Table S1. Batch Size and Cell Types Included

This table shows the number of cells selected for each batch size across various cell types in hematopoiesis dataset.

Batch Size	10	20	30	40	50
Undifferentiated	573	443	324	232	158
Neutrophil	490	383	262	184	120
Monocyte	382	323	257	208	126
Mast	53	2	2	2	1
Baso	49	24	2	2	1
Meg	12	4	1	0	0
Erythroid	10	7	0	0	0
Eos	5	2	1	1	1
Lymphoid	2	1	1	0	0
Ccr7_DC	1	1	0	0	0
Number of selected cells	1410	1180	870	720	500

Table S2. Multi-cell clones summary

This table displays the distribution of multi-cell clones grouped by size ranges in the hematopoiesis dataset.

Clone Size Range	Number of Clones	Percentage of Total
1 cell	37	2.8%
2-5 cells	793	60.6%
6-10 cells	225	17.2%
11-20 cells	145	11.1%
21-50 cells	91	7.0%
>50 cells	17	1.3%
Total (2 or more cells)	1271	97.2%
Total	1308	100%

Table S3. State-fate clones summary statistics

This table presents summary statistics of state-fate clones, including their total number and size distribution in hematopoiesis dataset.

Statistic	Value
Total number of state-fate clones	510
Median cells per clone	5
Mean cells per clone	9.3
Minimum cells per clone	2
Maximum cells per clone	92

Table S4. State-fate clones composition

This table shows the composition of state-fate clones across different time points in hematopoiesis dataset.

Clone Composition	Number of Clones	Percentage
Day 2 + Day 4 only	254	49.8%
Day 2 + Day 6 only	31	6.1%
Day 2 + Day 4 + Day 6	225	44.1%
Total	510	100%

Table S5. Average distance and p-value for each cell type.

The mean and standard deviation on the latent space were calculated for each cell type. This table displays the average distance and p-value for each cell type.

	Baso	Lymphoid	Mast	Meg	Monocyte	Neutrophil	Undifferentiated
Baso		3.31 (p=0.427)	3.70 (p=0.427)	4.71 (p=0.427)	2.89 (p=0.340)	2.76 (p=0.409)	2.34 (p=0.709)
Lymphoid	3.31 (p=0.427)		3.61 (p=0.273)	4.10 (p=0.241)	2.89 (p=0.111)	3.44 (p=0.161)	2.00 (p=0.686)
Mast	3.70 (p=0.427)	3.61 (p=0.273)		3.42 (p=0.970)	3.30 (p=0.997)	3.95 (p=0.875)	3.54 (p=0.270)
Meg	4.71 (p=0.427)	4.10 (p=0.241)	3.42 (p=0.970)		4.00 (p=0.916)	4.04 (p=0.779)	3.94 (p=0.240)
Monocyte	2.89 (p=0.340)	2.89 (p=0.111)	3.30 (p=0.997)	4.00 (p=0.916)		2.47 (p=0.074)	2.00 (p=0.000)
Neutrophil	2.76 (p=0.409)	3.44 (p=0.161)	3.95 (p=0.875)	4.04 (p=0.779)	2.47 (p=0.074)		1.88 (p=0.000)
Undifferentiated	2.34 (p=0.709)	2.00 (p=0.686)	3.54 (p=0.270)	3.94 (p=0.240)	2.00 (p=0.000)	1.88 (p=0.000)	

Table S6. TF Activity per time point for Different Cell Types.

The calculated activities of the defined TFs for each day were summed, and the totals for each date were then normalized, ensuring that the day with the highest sum equaled 1.00.

	Day					
**Cell Type**	**0**	**1**	**2**	**3**	**4**	**5**
Baso	0.76	0.80	0.63	0.94	1.00	0.86
Monocyte	1.00	0.79	0.67	0.68	0.80	0.98
Neutrophil	1.00	0.67	0.69	0.74	0.72	0.81

Table S7. Comparison of Methods for Differentiation Prediction.

A summary of various methods used for differentiation prediction, highlighting their basic idea, and whether they require or can use expression data, splicing kinetics, time series data, or lineage barcodes.
Symbols: ✓ Required, ● Available but not required.

**Methods**	**Basic Idea**	**Required Input**			
		**Expression**	**Splicing kinetics**	**Time Series**	**Lineage Barcodes**
Pseudotime	Similarity of expression	✓			
scVelo	Splicing kinetics	✓	✓		
VeloVAE	Splicing kinetics	✓	✓		
Waddington-OT	Optimal transport	✓		✓	
CoSpar	Sparse optimization	✓		✓	●
LineageVAE	VAE, Mixture of Experts	✓	●	✓	✓

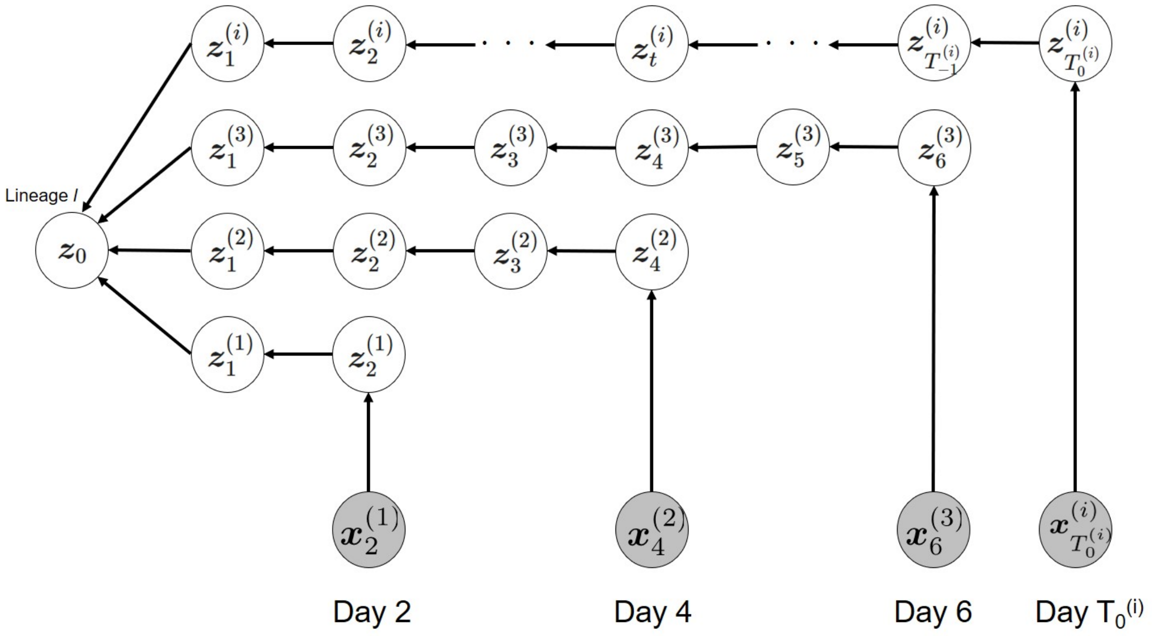

Fig. S1. LineageVAE graphical model. Shaded nodes x_t^i indicate observed data, and white node z_t^i symbolizes latent variables. Edges indicate dependencies. Variational inference is conducted backward in time from the observed data.

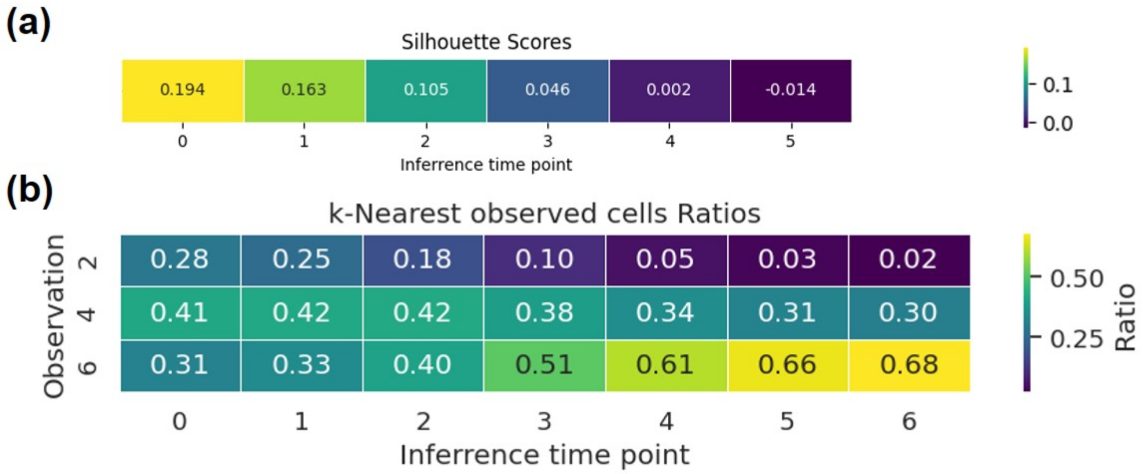

Fig. S2. Quantitative evaluation of time series inference. a Silhouette score for cells in each estimated time point for observed cells. **b** Ratio of observed cells that are k neighbors of the inferred cells.

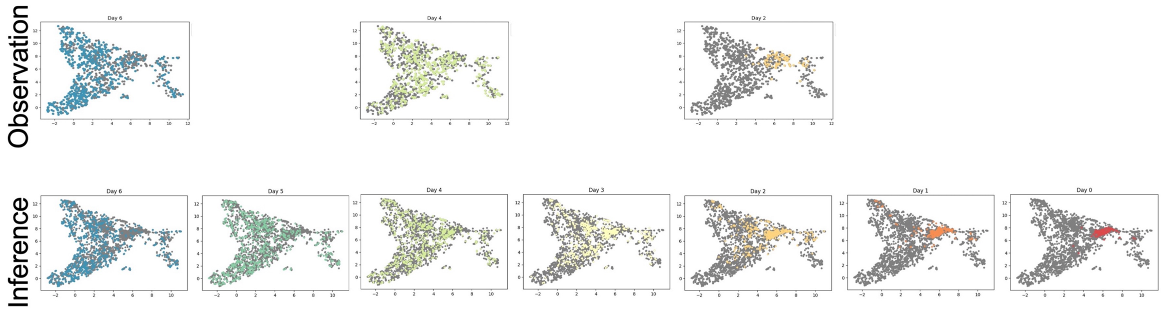

Fig. S3. Overview of the time series inference. (Upper) Observation cells and observation time points. (Lower) Inferred cells and time points.

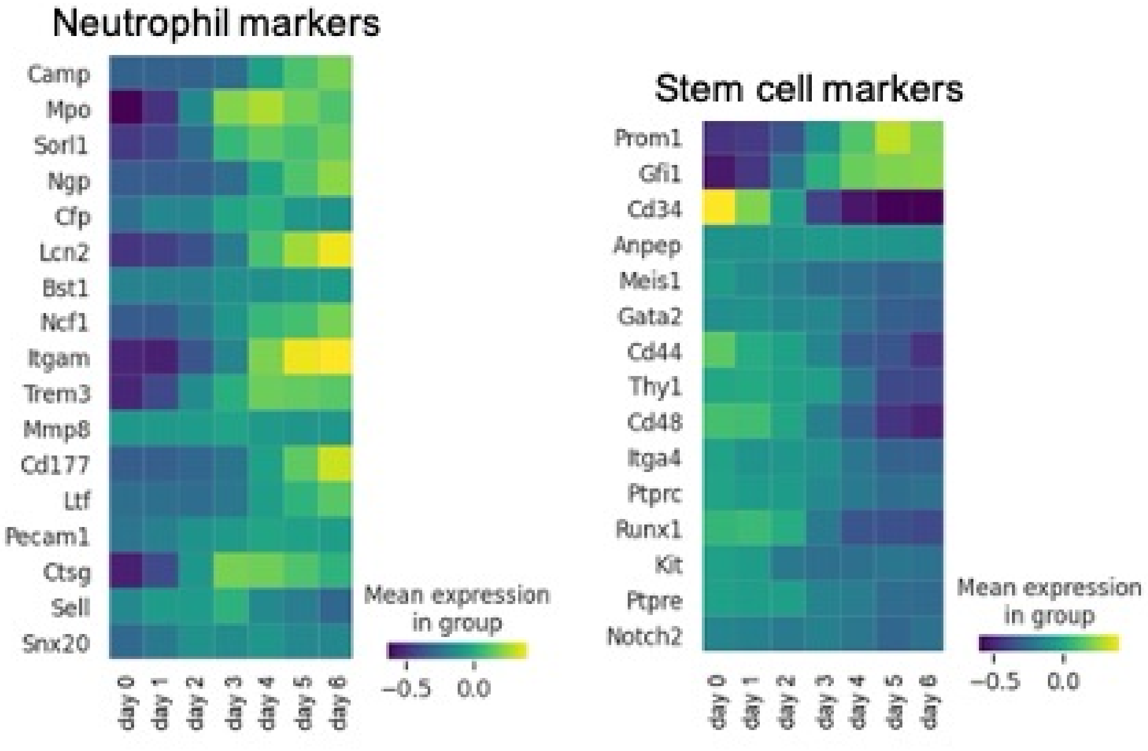

Fig. S4. Reconstruction of historical expression by LineageVAE. (Left) Inferred expression of Neutrophil differentiation markers at each time point in cells that differentiate into Neutrophil. (Right) Inferred expression of undifferentiated marker at each time point in cells that differentiate into Neutrophil.

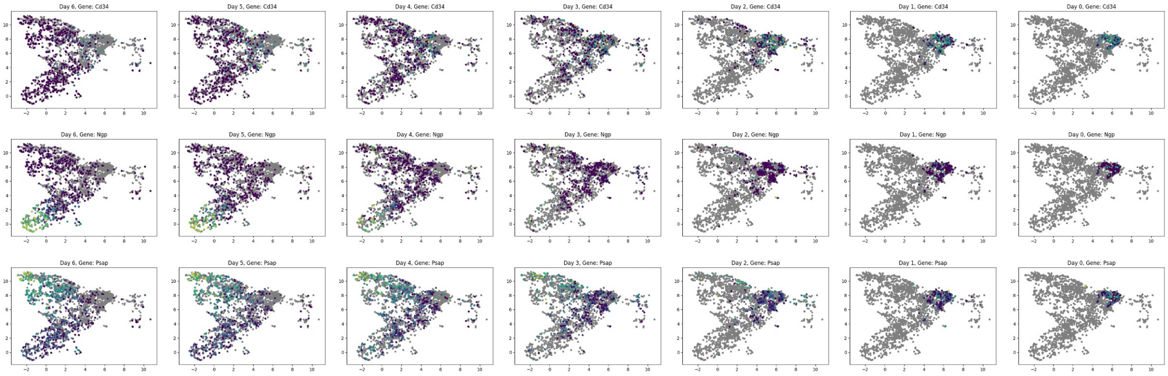

Fig. S5. Visualization of recovered gene expression on UMAP. (Upper) Neutrophil Marker: Ngp expression at each time point. (Middle) Monocyte Marker: Psap expression at each time point. (Lower) undifferentiated marker: Cd34 expression at each time point.

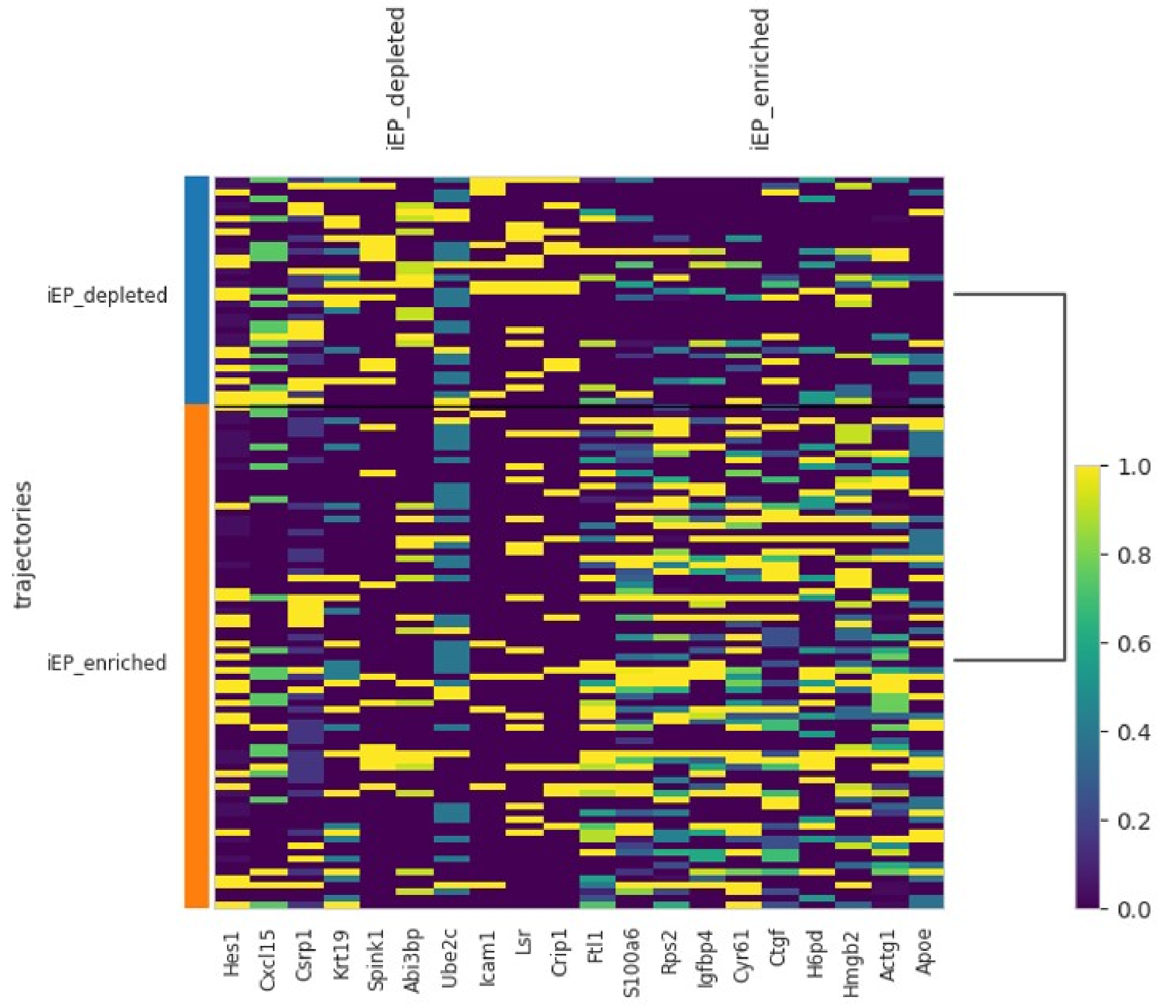

Fig. S6. Evaluation of progenitor bias by heatmaps. DEGs expression on day 0, which is experimentally unobservable after reprogramming induction.
