# Supplementary figures and images for: LineageVAE: reconstructing historical cell states and transcriptomes toward unobserved progenitors
Source: Bioinformatics. 2024 Aug 22;40(10):btae520. doi: 10.1093/bioinformatics/btae520 (PMC11494380; doi:10.1093/bioinformatics/btae520)

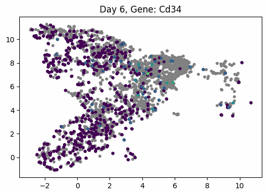

Supplement: btae520_Supplementary_Data [file btae520_supplementary_data.zip › cd34.gif]

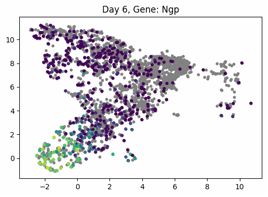

Supplement: btae520_Supplementary_Data [file btae520_supplementary_data.zip › ngp.gif]

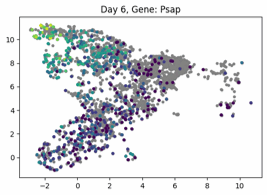

Supplement: btae520_Supplementary_Data [file btae520_supplementary_data.zip › psap.gif]

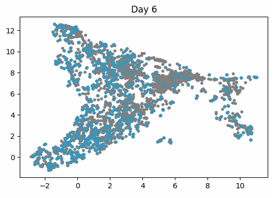

Supplement: btae520_Supplementary_Data [file btae520_supplementary_data.zip › state.gif]
